# Supplementary material for: Structural and functional characterization of the extended-diKH domain from the antiviral endoribonuclease KHNYN
Source: J Biol Chem. 2025 Feb 19;301(4):108336. doi: 10.1016/j.jbc.2025.108336 (PMC11997328; doi:10.1016/j.jbc.2025.108336)
Supplement: Supplementary Figures [file mmc1.pdf]

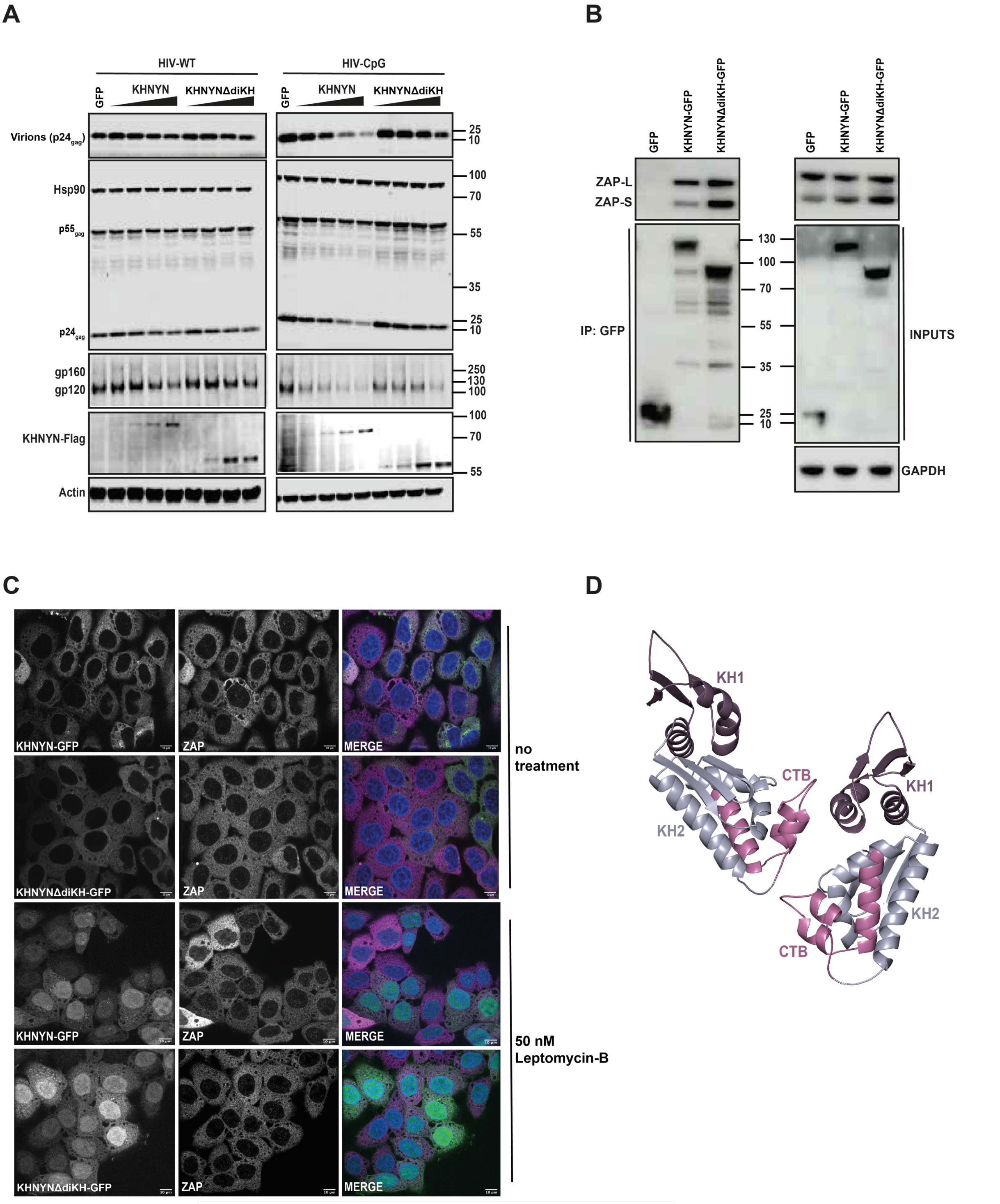

Figure S1. The KHNYN ex-diKH domain is required for antiviral activity but does not regulate the interaction with ZAP or KHNYN subcellular localization. (**A**) Representative western blotting corresponding to **Fig 1B**. (**B**) Representative ZAP-KHNYN co-immunoprecipitation experiment. Stable HeLa CRISPR KHNYN cells expressing GFP control, KHNYN-GFP or KHNYNΔdiKH-GFP were infected with either HIV-WT or HIV-CpG for 48 hours with MOI = 3. After infection, cells were lysed and immunoprecipitated using α-GFP antibodies. Samples were immunoblotted against GFP and ZAP. (**C**) Confocal

microscopy staining for GFP and ZAP in HeLa CRISPR KHNYN cells expressing KHNYN-GFP or KHNYNΔdiKH-GFP with no treatment or 50 nM of Leptomycin B for 4 hours at 37 °C. (**D**) The ex-diKH crystal structure asymmetric unit (ASU) relating to **Fig 1C**. The two ex-diKH protomers in the ASU are shown in cartoon representation. KH1, KH2 and CTB domains are colored purple, blue-grey and magenta respectively.

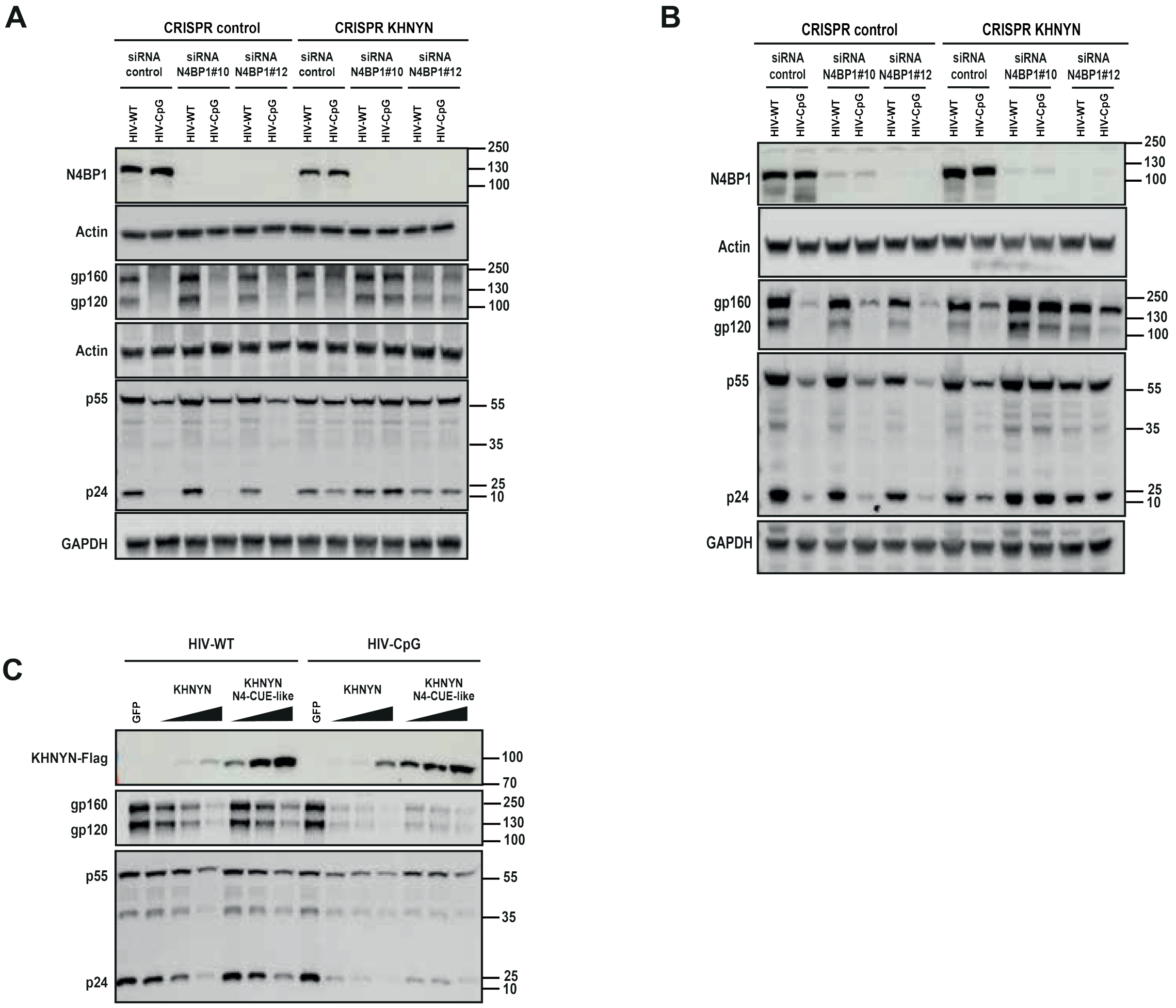

Figure S2. The KHNYN and N4BP1 ex-diKH domains are functionally equivalent. (**A**) Representative western blotting corresponding to **Fig 2B**. (**B**) Representative western blotting corresponding to **Fig 2C**. (**C**) Representative western blotting corresponding to **Fig 2F**.

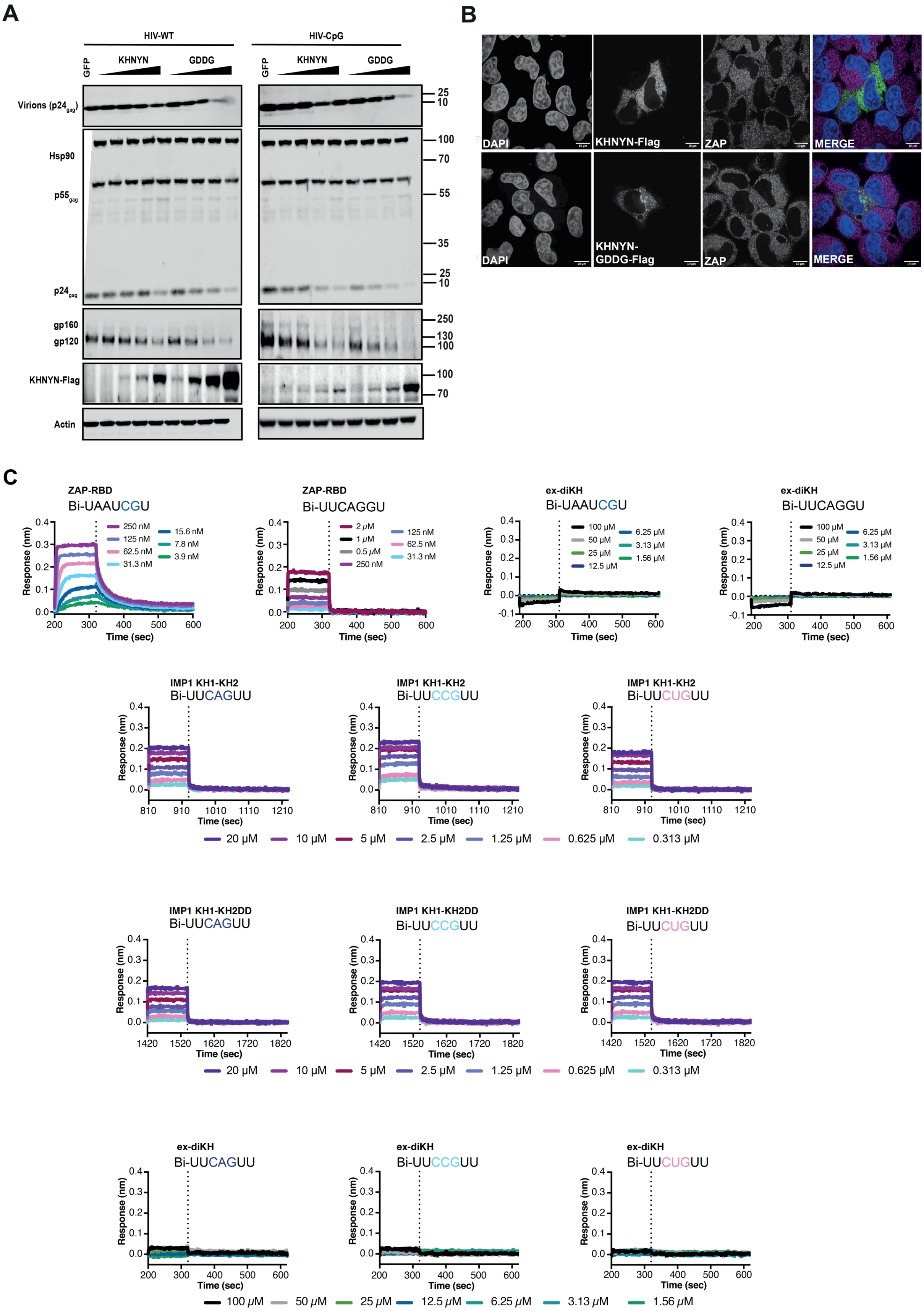
A
HIV-WT
HIV-CpG
GFP
KHNYN
GDDG
Virions (p24gag)
Hsp90
p55gag
p24gag
gp160
gp120
KHNYN-Flag
Actin
B
DAPI
KHNYN-Flag
ZAP
MERGE
KHNYN-GDDG-Flag
C
ZAP-RBD
Bi-UAAUCGU
Bi-UUCAGGU
ex-diKH
IMP1 KH1-KH2
Bi-UUCAGUU
Bi-UUCCGUU
Bi-UUCUGUU
IMP1 KH1-KH2DD
Response (nm)
Time (sec)

Figure S3. The KHNYN ex-diKH domain has no detectable RNA binding activity. (**A**) Representative western blotting corresponding to **Fig 3B**. (**B**) Confocal microscopy of HeLa CRISPR-KHNYN expressing either FLAG-tagged KHNYN or KHNYN(GDDG) mutant. Cells were stained for the FLAG-tag (green) and ZAP (magenta). (**C**) BLI analysis of ZAP RBD, KHNYN ex-diKH, IMP1(KH1-KH) and IMP1(KH1-KH2DD) RNA binding, relating to **Fig 3D-3E**. BLI association and dissociation response traces are shown from a single titration from triplicate BLI runs performed with immobilized biotinylated RNAs and varying concentration of proteins. Switchover from the association to the dissociation phase is indicated by the dashed line.
