# Supplementary material for: Structural and functional characterization of the extended-diKH domain from the antiviral endoribonuclease KHNYN
Source: J Biol Chem. 2025 Feb 19;301(4):108336. doi: 10.1016/j.jbc.2025.108336 (PMC11997328; doi:10.1016/j.jbc.2025.108336)
Supplement: Supplementary Tables [file mmc2.pdf]

Table S1. X-ray data collection and structure refinement statistics

	KHNYN-1(P8-G200) Dataset 1	KHNYN-1(P8-G200) Dataset 2 (STARANISO)
**Data collection**		
Space group	$P2_1$	$P2_1$
Cell dimensions		
a, b, c (Å)	28.87, 122.64, 44.57	28.83, 121.80, 44.55
α, β, γ	90, 108.90, 90	90, 108.55, 90
Wavelength (Å)	0.9795	0.9795
Resolution (Å)	61.32-2.18 (2.22-2.18)	60.90-2.20 (2.26-2.20)
Anisotropic diff. limits		
a*, b*, c* (Å)	-	2.68, 2.35, 2.04
Unique reflections	8927 (17)	10083 (183)
R_{meas}	0.066 (0.280)	0.188 (1.008)
R_{pim}	0.035 (0.198)	0.079 (0.448)
$CC_{1/2}$	0.996 (1.000)	0.995 (0.577)
I/σ(I)	24.3 (2.2)	7.5 (1.7)
Completeness		
Spherical (%)	58.7 (2.2)	73.3 (23.0)
Ellipsoidal (%)	-	90.8 (80.0)
Multiplicity	3.3 (1.1)	5.4 (4.8)
**Refinement**		
Resolution (Å)		60.9-2.20
R_{work}		0.196 (0.290)
R_{free}		0.258 (0.508)
R_{free} Reflections (%)		7.2
No. monomers/A.S.U.		2
No. atoms		
Protein		2764
Water		27
B-factors (Å)		
Wilson		27.22
Protein		32.94
Water		25.13
Average		32.86
R.m.s. deviations		
Bond lengths (Å)		0.009
Bond angles (°)		1.54
Chiral volumes (Å^3)		0.049
Ramachandran		
Favoured		96.52
Allowed		3.48
Outliers		0.00
PDB code		9HTS

†Values in parentheses refer to the highest resolution shell

Table S2. KHNYN diKH DALI search results

DALI results, Z score >8.0	Description	Z score	RMSD (Å)	Cα aligned	Seq ID (%)
6q3v	N4BP1 KH1KH2	23.9	1.8	174	37
6qey	IMP1 KH1KH2	9.6	5.9	69	13
2n8l	IMP1 KH3KH4(DD)	9.3	4.0	71	15
1ec6	Nova-2 KH3	9.0	3.0	68	6
2jvz	KSRP KH2KH3	8.9	2.4	66	12
5www	MEX3C KH1	8.9	2.9	79	16
4aim	PNPASE KH	8.8	9.2	76	5
2qnd	FMRP KH1KH2	8.3	1.7	63	16
2hh2	KSRP KH4	8.2	2.7	65	12
1tua	APE0754 KH	8.1	4.0	79	16
5wwx	MEX3C KH2	8.1	2.0	60	15

Table S3. EMSA RNA oligonucleotides

Name	Sequence
CpG-rich	FAM-5'-GCAACGACGACGCUAUUUUGCGCAUCAGACGCG-3'
CpG-depleted	FAM-5'-GCAACCACCACUCUAUUUUGUGCAUCAGAUGCU-3'
AU-rich	FAM-5'-GUAACUAAUACUCUAUUUUGUUAAUCAGAUAAU-3'
A rich	FAM-5'-AUAACAAAUACACUAAAAUACAAAUCAGAAAAU-3'
C-rich	FAM-5'-CUCCUCCCUACACUCCCCUCUCCCGCCGCCCCU-3'
U-rich	FAM-5'-GUUUCUUUUACUCUUUGUUACUUUUCAGUUAAU-3'

Supplementary Table S4. BLI RNA binding experiments

Protein	Sequence	K_D (μM) and 95 % CI
ZAP(1-227)	Bi-5'-UAAUCGU	0.03 (0.02-0.06)
ZAP(1-227)	Bi-5'-UUCAGGU	0.74 (0.54 – 1.01)
KHNYN-1 (8-200)	Bi-5'-UAAUCGU	n. m.
KHNYN-1 (8-200)	Bi-5'-UUCAGGU	n. m.
IMP1 (V194-N369)	Bi-5'-UUCAGUU	1.3 (0.7 – 2.2)
IMP1 (V194-N369	Bi-5'-UUCCGUU	0.7 (0.5 – 1.0)
IMP1 (V194-N369	Bi-5'-UUCUGUU	1.5 (0.9 – 2.4)
IMP1 (V104-N369, K294D, E295D)	Bi-5'-UUCAGUU	3.3 (2.0 – 5.4)
IMP1 (V104-N369, K294D, E295D)	Bi-5'-UUCCGUU	1.7 (0.9 – 3.0)
IMP1 (V104-N369, K294D, E295D)	Bi-5'-UUCUGUU	3.3 (1.9 – 5.6)
KHNYN-1 (8-200)	Bi-5'-UUCAGUU	n. m.
KHNYN-1 (8-200)	Bi-5'-UUCCGUU	n. m.
KHNYN-1 (8-200)	Bi-5'-UUCUGUU	n. m.

Table S5. Cloning primers

Construct	Orientation	Sequence (5'-3')
KHNYN(8-200)	FWD	CAGTACGACCCGGGGCAAGTCCGGATCGTTTTGC
KHNYN(8-200)	REV	GCTAGACTGAGCTCTCATCAACCTGAGCTTGCTTCTTGC
ZAP(1-227)	FWD	CAGGGACCCGGTATGGCAGATCCGGAAGTTTGTTG
ZAP(1-227)	REV	GGCACCAGAGCGTTAATTTTTCTGCATGTGTTTGCTGTTG
KHNYNΔdiKH	FWD	GAGGCGTCTAGTGGGCAG
KHNYNΔdiKH	REV	CATGGTGAATTCGGTAGATCTAATTCC
KHNYN N4-KH-GFP	FWD	CCGAGCTCGGATCCGCCACCATGGCGGCCCGGGCGGTGCTG
KHNYN N4-KH-GFP	REV	GGTGGCGGATCCGAGCTC
KHNYN N4-CUE-like	FWD	ATGGTGTTGGAT GATTACAAGGATGACGACGATAAG
KHNYN N4-CUE-like	REV	TCTCTGAGCTGG ACTACCTTTTTCCTCTTCTCTCC
KHNYN N4-CUE-like	FWD	GAAAAAGGTAGT CCAGCTCAGAGATCTTCTGCAG
KHNYN N4-CUE-like	REV	ATCCTTGTAATC ATCCAACACCATGGCAGAAAG
KHNYN(GDDG)	FWD	CAAACTGCACTGCATCTTTCTGGGAGACGACGGCTTCTTCCTTGACTGCCTGGCCTG
KHNYN(GDDG)	REV	5'CAGGCCAGGCAGTCAAGGAAGAAGCCGTCGTCTCCCAGAAAGATGCAGTGCAGTTTG
KHNYN(R33A)	FWD	CCATGTGGAGGCCATCTTCAGCGTGGG
KHNYN(R33A)	REV	GGCTGCTGTTCCCGAACC
KHNYN(R65A)	FWD	AAACGCCAGCGCAGCCAAGGAGTACCTG
KHNYN(R65A)	REV	TCCTTGGGGCCCTCCAGC

Table S6. Protein purification buffers

Protein	Buffer Name	Buffer Composition
KHNYN(8-200)	Lysis Buffer K	100 mM BTP-HCl pH 8.5, 150 mM NaCl, 0.5 mM TCEP, 5% w/v glycerol, 0.2% v/v Triton X-100, 1x cOmplete EDTA-free protease inhibitor cocktail tablet per 50 mL (Roche) and 0.4 units/mL benzonase nuclease (Millipore)
ZAP(1-227)	Lysis Buffer Z	100 mM BTP-HCl pH 6.5, 800 mM NaCl, 0.5 mM TCEP, 5% w/v glycerol, 10 mM $MgCl_2$, 1x cOmplete EDTA-free protease inhibitor cocktail tablet per 50 mL (Roche), 2.5 units/mL universal nuclease (Pierce) and 1.7 units/mL salt active nuclease (Sigma)
KHNYN(8-200)	Wash K1	100 mM BTP-HCl pH 8.5, 150 mM NaCl, 0.5 mM TCEP
KHNYN(8-200)	Wash K2	100 mM BTP-HCl pH 8.5, 1 M NaCl, 0.5 mM TCEP
ZAP(1-227)	Wash Z1	100 mM BTP-HCl pH 6.5, 800 mM NaCl, 0.5 mM TCEP, 5% w/v glycerol
KHNYN(8-200)	Elution buffer KE	100 mM BTP-HCl pH 8.5, 150 mM NaCl, 0.5 mM TCEP, 50 mM biotin
ZAP(1-227)	Elution buffer ZE	100 mM BTP-HCl pH 6.5, 800 mM NaCl, 0.5 mM TCEP, 5% w/v glycerol, 50 mM biotin
KHNYN(8-200)	Gel filtration KGF	50 mM BTP-HCl pH 8.5, 150 mM NaCl, 0.5 mM TCEP
ZAP(1-227)	Low Salt	100 mM BTP-HCl pH 6.5, 250 mM NaCl, 0.5 mM TCEP, 5% w/v glycerol
ZAP(1-227)	IEX	100 mM BTP-HCl pH 6.5, 125 mM NaCl, 0.5 mM TCEP, 5% w/v glycerol
ZAP(1-227)	Gel filtration ZGF	50 mM BTP-HCl pH 6.5, 200 mM NaCl, 0.5 mM TCEP, 5% w/v glycerol
